# Characterization and initial demonstration of *in vivo* efficacy of a novel heat-activated metalloenediyne anti-cancer agent

**DOI:** 10.1080/02656736.2021.2024280

**Published:** 2022

**Authors:** Joy Garrett, Erin Metzger, Mark W. Dewhirst, Karen E. Pollok, John J. Turchi, Isabelle C. Le Poole, Kira Couch, Logan Lew, Anthony Sinn, Jeffrey M. Zaleski, Joseph R. Dynlacht

**Affiliations:** a Department of Radiation Oncology, Indiana University School of Medicine, Indianapolis, IN, USA;; b Department of Chemistry, Indiana University, Bloomington, IN, USA;; c Department of Radiation Oncology, Duke University School of Medicine, Durham, NC, USA;; d In Vivo Therapeutics Core, Indiana University Melvin and Bren Simon Comprehensive Cancer Center, Indiana University School of Medicine, Indianapolis, IN, USA;; e Department of Medicine, Indiana University School of Medicine, Indianapolis, IN, USA;; f Department of Dermatology, Northwestern University, Chicago, IL, USA

**Keywords:** Metalloenediyne, melanoma, thermal activation, hyperthermia, thermal ablation

## Abstract

**Background:**

Enediynes are anti-cancer agents that are highly cytotoxic due to their propensity for low thermal activation of radical generation. The diradical intermediate produced from Bergman cyclization of the enediyne moiety may induce DNA damage and cell lethality. The cytotoxicity of enediynes and difficulties in controlling their thermal cyclization has limited their clinical use. We recently showed that enediyne toxicity at 37 °C can be mitigated by metallation, but cytotoxic effects of ‘metalloenediynes’ on cultured tumor cells are potentiated by hyperthermia. Reduction of cytotoxicity at normothermia suggests metalloenediynes will have a large therapeutic margin, with cell death occurring primarily in the heated tumor. Based on our previous *in vitro* findings, FeSO_4_-PyED, an Fe co-factor complex of (*Z*)-N,N^׳^-bis[1-pyridin-2-yl-meth-(*E*)-ylidene]oct-4-ene-2,6-diyne-1,8-diamine, was prioritized for further *in vitro* and *in vivo* testing in normal human melanocytes and melanoma cells.

**Methods:**

Clonogenic survival, apopotosis and DNA binding assays were used to determine mechanisms of enhancement of FeSO_4_-PyED cytotoxicity by hyperthermia. A murine human melanoma xenograft model was used to assess *in vivo* efficacy of FeSO_4_-PyED at 37 or 42.5 °C.

**Results:**

FeSO_4_-PyED is a DNA-binding compound. Enhancement of FeSO_4_-PyED cytotoxicity by hyperthermia in melanoma cells was due to Bergman cyclization, diradical formation, and increased apoptosis. Thermal enhancement, however, was not observed in melanocytes. FeSO_4_-PyED inhibited tumor growth when melanomas were heated during drug treatment, without inducing normal tissue damage.

**Conclusion:**

By leveraging the unique thermal activation properties of metalloenediynes, we propose that localized moderate hyperthermia can be used to confine the cytotoxicity of these compounds to tumors, while sparing normal tissue.

## Introduction

Enediynes are a class of natural products that were first recognized for their potent anti-tumor activities in the late 1980s. Since then, several derivatives have been synthesized, but common to all enediynes are two acetylenic groups conjugated to a double bond commonly situated within a nine- or ten-membered ring [1]. It is this uniquely strained structure that confers the cytotoxic tendencies of these compounds, as an appropriate chemical or physical trigger will induce the enediyne core to undergo Bergman cyclization. This cycloaromatization results in the production of a benzenoid 1,4-diradical intermediate that may go on to abstract H-atoms from DNA, resulting in strand breakage. While the DNA-damaging aspects of enediynes are tantalizing, their clinical utility has been limited due to challenges in controlling the initiation of diradical formation; the mild activation temperatures for enediyne cyclization (generally less than 37 °C) lead to toxicity. Combined with difficulties of synthetic accessibility, these factors discouraged their further development.

The late 1980s also saw interest in hyperthermia as a cancer modality peak with the hope that a better understanding of the biological effects of moderate hyperthermia treatments (~41–45.5 °C) might improve clinical outcomes of chemotherapy and radiotherapy. Heat inhibits repair of DNA damage, among other cellular and biochemical processes, and has been shown to sensitize tumor cells to DNA damaging chemicals and ionizing radiation (IR) [2]. Potentiation of effects of some chemotherapy agents by thermal therapy may be due to increased drug influx or decreased efflux, which often results in a greater intracellular concentration of the drug, leading to a greater induction of DNA damage [3–6]. Unfortunately, interest in hyperthermia waned after most clinical trials in the United States failed to show clinical benefit, due in part to technical limitations for delivering adequate heat doses to tumors [7].

Coincidentally, there has been a resurgence of interest in both enediynes and hyperthermia. For hyperthermia, interest has been renewed as technology for accurate delivery of heat doses and standards for quality assurance advanced [8–11], and after many clinical trials for several different malignancies indicated efficacy in tumor control and palliative care [12–15]. Though some limitations remain, moderate hyperthermia continues to be evaluated for its ability to enhance effects of chemotherapy, radiotherapy (including proton therapy), chemoradiotherapy, and more recently, immunotherapy [12,13,16,17]. There has also been a recent increase in clinical applications of thermal ablation, which involves the local application of high temperatures (usually ≥50° C). For example, percutaneous thermal ablation is now a first-line option for the treatment of some liver tumors [18]. It is also used to treat deep-seated bone, kidney, liver, and lung tumors, and has been proposed for treatment of early-stage breast cancer [19, and references therein).

With regard to enediynes, there has been renewed interest in the development of novel constructs as potential therapeutic agents with advances in the use of metal ions to control activation through geometric structure changes [20,21]. Indeed, the binding of metals to an enediyne can greatly modulate their cyclization temperature compared to an uncomplexed enediyne [22–27].

Recently, we synthesized a suite of metal-mediated radical generators based on the tetradentate ligand (*Z*)-*N*,*N*-bis[1-pyridin-2-yl-meth(*E*)-ylidene]oct-4-ene-2,6-diyne-1,8-diamine (PyED) which can undergo Bergman cyclization within 3 h at room temperature and form a 1,4-benzenoid diradical upon chelation with metals [28,29]. We have been characterizing the biological effects of these compounds on tumor cells at physiological and hyperthermic temperatures. We found that treatment of tumor cells with these metallated enediyne motifs results in a significant enhancement of cytotoxicity when cells are exposed to the compounds at elevated temperatures, but that little or no cytotoxicity results when cells are treated at 37° C [30]. Heating melanoma and breast cancer cells at 42.5° C during treatment with metalloenediynes increased apoptosis and resulted in inhibition of double strand break (DSB) repair [31,32]. In the present study, we provide additional evidence for the mechanism of enhanced cytotoxicity of metalloenediynes when melanoma cells are treated at hyperthermic temperatures, and demonstrate differential effects in cell killing between human melanocytes and melanoma cells. We also now report the first demonstration of *in vivo* efficacy of a novel metalloenediyne compound.

Heterogeneous or otherwise insufficient destruction of tumors following thermal ablation due to sublethal heating at the tumor periphery could result in recurrence and/or metastasis [33]. Thus, our studies should result in increased interest in metalloenediynes for clinical development in combination with thermal ablation as well as moderate hyperthermia, since metalloenediynes activated by moderate heating at the periphery of the ablation zone could potentially enhance tumor cell death and reduce the probability of tumor regrowth.

## Materials and methods

### Syntheses of compounds

**Figure 1.**
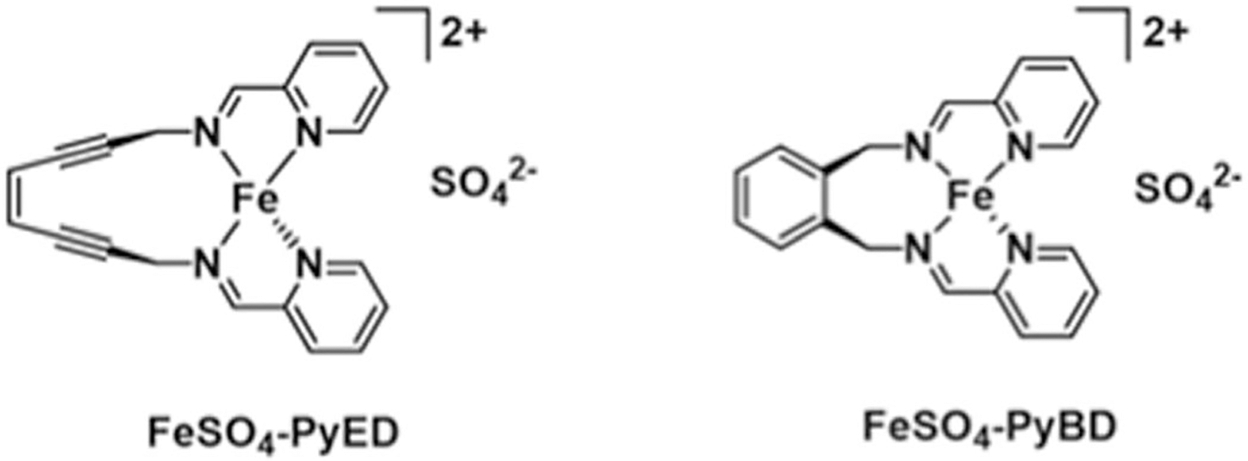
Structures of FeSO_4_-PyED and FeSO_4_-PyBD. FeSO_4_-PyED is capable of undergoing Bergman cyclization [19], while FeSO_4_-PyBD is a cyclized ‘radical control’ version of FeSO_4_-PyED that does not undergo Bergman cyclization or form diradical intermediates.

Syntheses and metal complexations were performed under a nitrogen atmosphere using standard Schlenk and dry box techniques and chemicals of the highest purity. The ligands (*Z*)-N,N’-bis[1-pyridin-2-yl-meth-(*E*)-ylidene]oct-4-ene-2,6-diyne-1,8-diamine and pyridin-2-ylmethyl-(2-{[(pyridine-2-ylmethylene)-amino]-methyl}-benzyl)-amine (PyED and PyBD, respectively) and metal complexes were prepared using previously published syntheses [29]. Briefly, a cooled solution of PyED or PyBD (1 eq) in methanol was added to a stirring solution of $\text{Fe}\left(\text{II}\right){\text{SO}}_{4^{\cdot }}\times {\text{H}}_{2}\text{O}$ (1 eq) in methanol and stirred for 4 h at 0 °C. The solvent from each solution was removed *in vacuo* at 0 °C, and diethyl ether was then added to the crude products and stirred for 2 h. Finally, the suspension was filtered to yield the solid products FeSO_4_-PyED and FeSO_4_-PyBD.Structures of these compounds are shown in Figure 1.

### Cell lines

U-1 melanoma cells were cultured in McCoy’s 5 A Medium (Mediatech Inc./Corning Inc., Manassas, VA) with 10% iron-supplemented calf serum (ISCS)(Hyclone Laboratories, Logan, UT). PIG1 human melanocytes were cultured in M254 medium supplemented with Human Melanocyte Growth Supplement-2, L-glutamine (200 mM), and Antibiotic-Antimycotic (Life Technologies, Grand Island, NY). Authentication of U-1 cells was performed at IDEXX Biolytics (Columbia, MO). Isolation, cultivation and characterization of PIG1 cells were described previously by Le Poole et al. [34]. Briefly, neonatal human foreskin melanocytes isolated from skin samples were exposed to the LXSN16E5E7 retroviral vector, containing the E6 and E7 genes of human papilloma virus type 16 (HPV 16). Transfectants were selected based on geneticin resistance and genetic homogeneity ensured through subcloning. While PIG1 cells have growth potential at least twice that of primary normal human melanocytes, they retain melanocytic properties that are otherwise normal without an extensively altered phenotype. Importantly, they retain a 2n karyotype and will not form tumors in nude mice. Both cell lines were maintained in logarithmic growth in an incubator at 5% CO_2_ and 37 °C.

### Clonogenic cell survival assay

Three days prior to experiments, 2.5 × 10^5^ U-1 melanoma cells were plated into T-25 flasks. Cells were allowed to reach ~90% confluency by the day of each experiment. Compounds were dissolved in sterile water and the cells treated with vehicle (water) or various concentrations of drug in the vehicle (1.5% by volume). After 1 h of treatment at either 37 °C or 42.5 °C in a precision-controlled water bath, the media containing drug or vehicle was removed and the cells were washed with fresh media. Cells were then trypsinized, counted, and 200–5000 cells (depending on the treatment) were plated into T-25 flasks. Flasks were then placed inside an incubator for 11 days to allow for colony formation. Colonies were fixed, stained, and counted. Only colonies which contained greater than 50 cells were included in the survival analysis. The mean plating efficiency for untreated control cells (no vehicle) for all experiments was 37.8% [standard error of the mean (SEM)=7%]. The mean plating efficiencies were 44.5% (SEM=±10.7%) for cells treated with vehicle (e.g., 0 μM of drug) for 1 h at 37° C, and 22.9% (SEM=±8.1%) for cells treated with vehicle (e.g., 0 μM of drug) for 1 h at 42.5° C. The surviving fractions of cells from the various drug treatments were calculated after normalizing to the plating efficiency of cells treated only with vehicle at the respective temperature.

### Compound-DNA binding assay

The DNA binding ability of FeSO_4_-PyED was assessed using a competitive DNA intercalation assay as described previously [31,35,36]. Briefly, salmon sperm DNA [10 ng/μL, (Thermo Fisher, Waltham, MA) in 25 mM MOPS (pH 6.5)] was incubated with various concentrations of FeSO_4_-PyED in water in a 96 well plate. This mixture was incubated at room temperature for 5–7 min before the addition of SYBR green (Sigma-Aldrich, St. Louis, MO). Fluorescence was measured using a BioTek Synergy H1 Hybrid Multi-mode Microplate Reader and BioTek Gen5 reader software (Winooski, VT) (excitation wavelength = 485 nm; emission wavelength = 528 nm; read height of 7 mm).

### Apoptosis assay

The apoptotic fraction of PIG1 cells was determined using an Annexin V-EGFP Apoptosis Kit (BioVision Inc., Milpitas, CA) as described previously [32]. Four days prior to experiments, PIG1 cells from stock culture flasks were dislodged using TripLE Express (Life Technologies Corporation, Grand Island, NY), centrifuged, resuspended in fresh medium, counted, plated into T25 flasks and allowed to grow logarithmically. On the day of experiments, cells were treated with either vehicle (water) or FeSO_4_-PyED at either 37° C or 42.5° C for 1 h and then incubated in fresh media for various times before being processed for determination of apoptotic fraction. Cells were first dislodged and counted. Then, 500,000 PIG1 cells per sample were resuspended in binding buffer, Annexin V-EGFP, and propidium iodide (PI) before being analyzed for green and red fluorescence using a BD LSR4 flow cytometer (BD Biosciences, San Jose, CA). Results were analyzed using the FlowJo program (BD, Ashland, OR).

### Determination of tumor growth inhibition

*In vivo* studies were approved by the Indiana University (IU) School of Medicine Institutional Animal Care and Use Committee. NOD.Cg-Rag1tm1Mom Il2rgtm1Wjl/SzJ (NRG) male mice (6–8 weeks old) were obtained from the on-site breeding colony of the *In Vivo* Therapeutics Core of the IU Simon Comprehensive Cancer Center (IUSCC). Animals were maintained under pathogen-free conditions on Teklad Uniprim Medicated Diet (TD 06596, Harlan Laboratories, Indianapolis, IN) with *ad libitum* access to sterile tap water under a 12-h light-dark cycle at 22–24 °C. C32 human melanoma cells (ATCC #CRL-1585) were expanded in DMEM (Gibco, ThermoFisher Scientific, Waltham, MA) with 10% ISCS, 1% HEPES, 1% sodium pyruvate, and 1% NEAA, and subcultured every 2–3 days. Cells were tested for mycoplasma before being implanted (see below) and the tests were always negative. C32 cells were authenticated at IDEXX Biolytics. After 10 days of cell expansion, 5 × 10^6^ cells in a 1:1 mixture with Corning Matrigel Growth Factor Reduced Basement Membrane Matrix (ThermoFisher Scientific, Waltham, MA) were implanted in the right hind limb of each mouse. On the 11th day post-implantation, C32 tumor volumes averaged 167 mm^3^. Mice were randomized into either ‘Vehicle Only’, ‘Vehicle + Heat’, ‘FeSO_4_-PyED’, or ‘FeSO_4_-PyED + Heat’ groups [tumor volumes (mean ± SEM) for each group were 163 ± 12.5 mm^3^, 174 ± 26.0 mm^3^, 165 ± 14.5 mm^3^ and 166 ± 15.4 mm^3^, respectively]. Volumes were calculated using the following formula: length × width × width × 0.5. The 11th day post-implantation was considered Day 0 of the experiment, as this was the day mice were dosed with FeSO_4_-PyED mixed in saline at 10 mg/mL and exposed to hyperthermia or normothermia. Mice received two injections of FeSO_4_-PyED (or vehicle) in the right hind limb and one IP injection. The maximum tolerated dose was determined to be 50 mg/kg. Mice received a total dose of 30 mg/kg in the first experiment and 50 mg/kg in experiments thereafter. For all experiments, one injection in the leg was directly intratumoral (IT) and administered to the center of the tumor (representing 12.5–20% of the total dose), while the other injection was administered subcutaneously at the periphery of the tumor (12.5–20% of the total dose). The IP injection represented 60–75% of the total dose, depending upon whether the mouse received 30 mg/kg or 50 mg/kg. Since both the 30 and 50 mg/kg dosing strategies resulted in similar results, data from experiments involving both dosing strategies were pooled.

Thirty minutes after injections, mice were anesthetized (100 mg/kg ketamine, 2.2 mg/kg acepromazine, 0.45 mg/kg atropine). Each mouse was placed in a restrainer that allowed the tumor-bearing right hind limb to be held away from the rest of the body. A plastic bag was secured around the restrainer to protect the hind limb from water after submersion into a water bath. The restrainer was attached to a holder and the right hind limb submerged in either a 34 °C (control) or 43.5 °C water bath for 1 h. Tumor temperatures (at the periphery and center of the tumor) were monitored in pilot studies using a hypodermic needle microprobe (Physitemp Instruments, Inc., Clifton, NJ), and a rectal probe (Physitemp Instruments) was used to monitor the body temperature of mice with legs submerged in the 43.5 °C water bath to ensure they did not rise above 38.5 °C (data not shown). In pilot testing, submersion of the leg into a 34 °C water bath raised the tumor temperature to 33.5 ± 0.2 °C within 4 min, and it remained stable throughout the remaining treatment time (data not shown). Submersion of the leg into a 43.5 °C water bath raised the temperature of the tumor periphery to an average temperature of 41.0 °C within 4 min, and both the tumor center and tumor periphery reached the target temperature of 42.5°C±0.5 °C within 20 min of submersion and remained at the target temperature for the remainder of the treatment time (the periphery was usually 0.3 °C greater than the center). The rectal temperature of mice with hind limbs submerged in a 34 °C bath averaged 32.3 °C within 20 min, and was stable (32.3±0.5 °C) throughout the remainder of the submersion. The rectal temperature of mice with hind limbs submerged in a 43.5 °C bath was 37.0 ± 1.5 °C after 20 min of submersion. However, if the rectal temperature approached 38.5 °C during heating of the hind limb, a fan was used to reduce the body temperature to ~37 °C and regulate it via air cooling. Occasionally, an additional ~33% of the original ketamine cocktail dose was administered if a mouse showed signs of the anesthesia wearing off during hind-limb submersion. After 1 h in the water bath, mice were removed from the restrainers and allowed to recover from the anesthesia by placing them in a plastic cage placed on a warming pad. Once ambulatory, mice were returned to the animal facility.

For 2 weeks following the treatments, tumors were monitored daily and measured 3 times per week. Digital calipers were used for measuring tumor volume. Because the experiment was repeated multiple times and the starting tumor volumes varied within and between experiments, the percent growth was calculated from the Day 0 starting volume in order to standardize the data. On the final day of the study (day 14 post-treatment), tumor volume measurements were taken and each mouse was sacrificed using CO_2_ inhalation and cervical dislocation. Tumors were removed and weighed after mice were sacrificed.

## Results

We have previously shown that the cytotoxicity of several metalloenediyne derivatives of PyED is greatly enhanced when U-1 melanoma and MDA-231 breast cancer cells are treated with the compounds at elevated temperatures [31,32]. One such compound, FeSO_4_-PyED, was found to be relatively nontoxic to cells after treatment with high concentrations at 37 °C, but cell killing was significantly enhanced when cells were treated with drug during hyperthermia treatment. Because of its low cytotoxicity after treatment at 37 °C and the magnitude of enhancement of cytotoxicity when cells were treated at hyperthermic temperatures compared to other compounds [32], we chose to further characterize FeSO_4_-PyED *in vitro* and ultimately test the efficacy of the compound *in vivo*.

**Figure 2.**
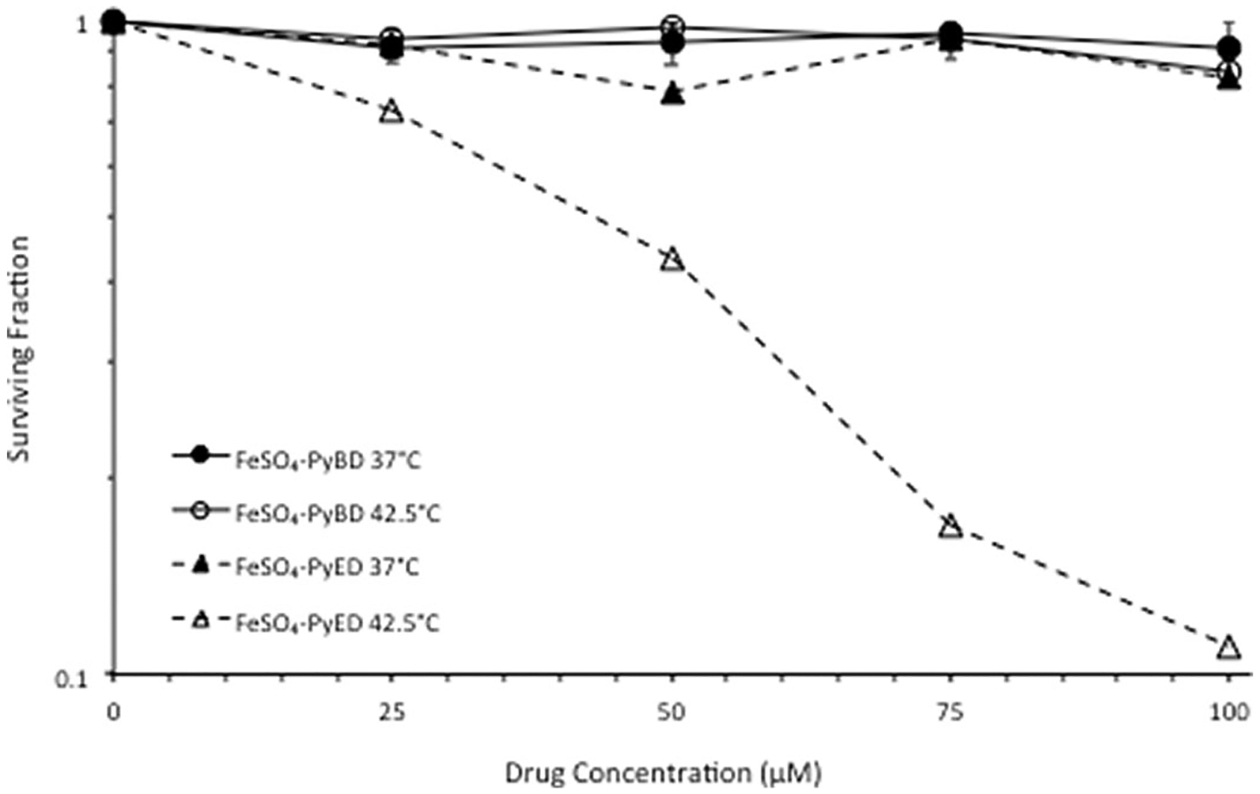
Effect of the diradical-generating enediyne moiety on enhancement of metalloenedyine cytotoxicity under hyperthermic conditions. U-1 cells were treated with various concentrations of FeSO_4_-PyED or its nonradical-generating analog, FeSO4-PyBD, for 1 h at 37 °C or 42.5 °C, and then plated for assessment of clonogenic survival and determination of surviving fraction. For FeSO_4_-PyBD data, error bars represent the standard error of the mean (SEM) from two experiments; FeSO_4_-PyED data are from one experiment that is representative of data from previously published experiments [32].

As shown in Figure 2 and in Garrett et al. [32], exposure to various concentrations of FeSO_4_-PyED up to 100 μM at 37 °C resulted in little to no killing of U-1 melanoma cells. However, enhancement of cell killing was concentration-dependent when cells were treated with the drug at 42.5 °C. To determine the relationship between Bergman cyclization and cytotoxicity at physiological and supra-physiological temperatures, we treated U-1 cells with the non-reactive cyclized analog FeSO4-PyBD at 37° or 42.5 °C. Treatment of cells with FeSO4-PyBD at 37 °C resulted in minimal or no cytotoxicity. Cytotoxicity was not enhanced when cells were treated with FeSO4-PyBD at 42.5 °C (that is, cell survival curves generated for cells treated at 37° or 42.5 °C with FeSO_4_-PyBD were superimposable).

**Figure 3.**
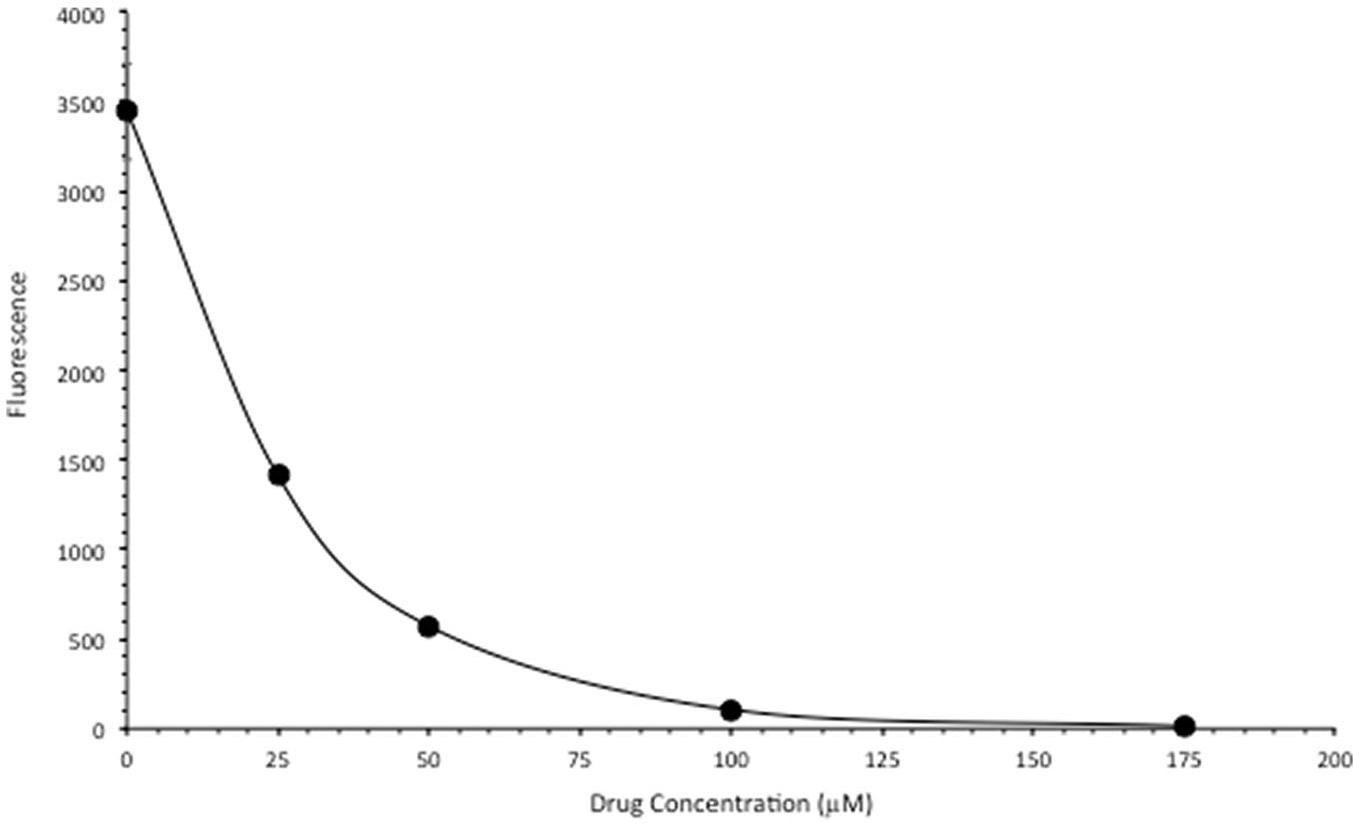
FeSO_4_-PyED is a DNA-binding metalloenediyne. SYBR Green-labeled salmon sperm DNA was incubated in either water or various concentrations of FeSO_4_-PyED. Loss of dye fluorescence is reflective of binding (intercalation) of FeSO_4_-PyED to DNA. Error bars represent the SEM for triplicate determinations.

We next tested the intercalative DNA binding capacity of FeSO_4_-PyED using a fluorescence displacement assay. Salmon sperm DNA was incubated at room temperature in the presence of either water or various concentrations of FeSO_4_-PyED. When SYBR Green is added, it binds to DNA and fluoresces, but the fluorescence decreased as the concentration of the FeSO_4_-PyED increased (Figure 3). These data demonstrate that FeSO_4_-PyED binds to DNA in a concentration-dependent manner, thus inhibiting SYBR Green from binding to the DNA.

**Figure 4.**
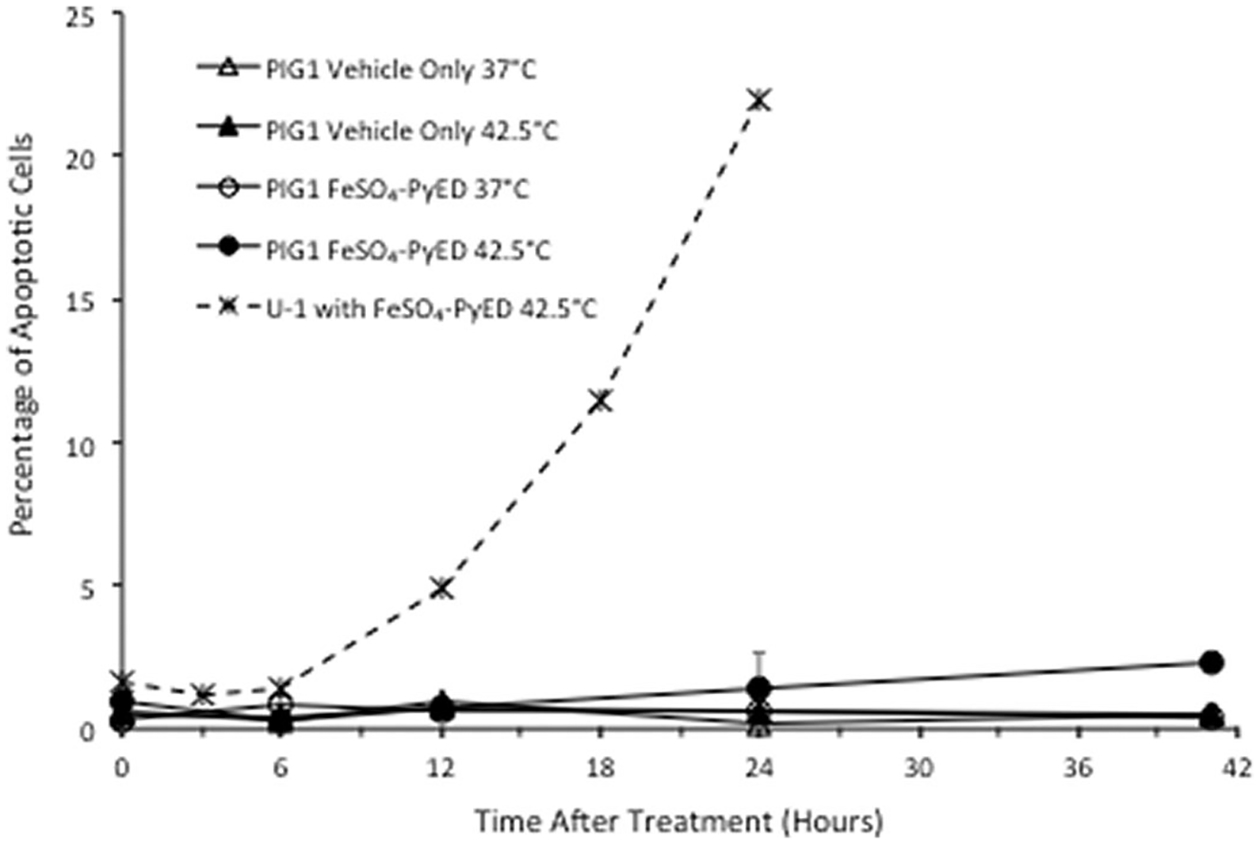
Differential effect of hyperthermia on apoptosis induced by FeSO_4_-PyED in U-1 melanoma and PIG1 melanocytes. Cells were incubated with 100 μM FeSO_4_-PyED or vehicle (1.5% water) for 1 h at 37 °C or 42.5 °C and then incubated at 37 °C with fresh media for various times. Apoptotic fractions were determined from bivariate plots of Annexin V-EGFP and PI fluorescence after treatments (not shown). The dashed line is representative of data from previously-published experiments with FeSO_4_-PyED on U-1 melanoma cells. Error bars represent SEM for 2–3 experiments.

Neither hyperthermia alone nor treatment with metalloenediynes at 37 °C induce apoptosis in U-1 melanoma cells, but populations of cells treated with metalloenediynes during heating undergo extensive apoptosis, as shown previously [31,32]. As further validation, when U-1 cells were treated with 100 μM FeSO_4_-PyED for 1 h at 42.5 °C and then incubated for various times after treatment prior to flow cytometric analysis of the apoptotic fraction, ~5–22% of cells were undergoing apoptosis at the time of measurement within 24 h post-treatment (Figure 4). Thus, the enhancing effect of hyperthermia on FeSO_4_-PyED cytotoxicity of melanoma cells appears to be attributed to an enhancement of apoptotic death. Similar to U-1 melanoma cells, PIG1 immortalized normal human melanocytes treated with 100 μM FeSO_4_-PyED or hyperthermia alone (1 h at 42.5 °C) did not undergo apoptosis (≤1% of cells were found to be apoptotic throughout the 41 h of observation post-treatment). However, unlike what was observed for U-1 cells, the apoptotic fraction of PIG1 cells that had been heated during treatment with FeSO_4_-PyED was not significantly different from PIG1 cells 24 h after treatment with vehicle only at 37 °C (the apoptotic fraction did not exceed ~2% over the entire period of observation post-treatment).

**Figure 5.**
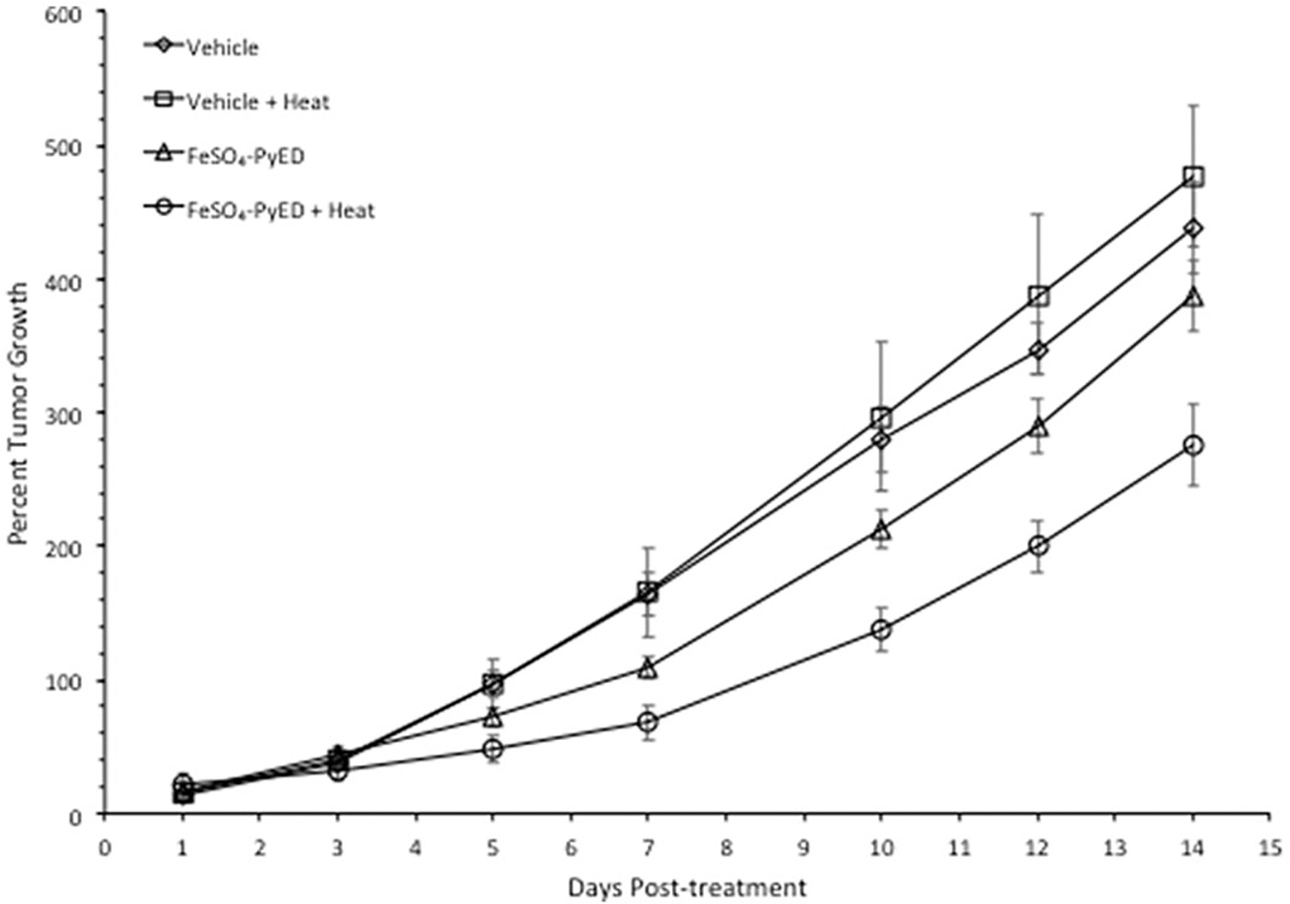
Treatment of C32 melanomas with FeSO_4_-PyED during heating inhibits tumor growth in a xenograft mouse model: Tumor volume measurements. Tumors were grown in the right hind limb of NRG mice, which were then injected with drug or vehicle and maintained for 1 h at either 33.5° or 42.5 °C by immersing the hind limb in a 34° or 43.5 °C water bath, respectively. Over the 14-day period of observation after treatment, tumor volume every other day was calculated and compared to the tumor volume on Day 0 (pre-treatment) to determine percent growth of each tumor. For each day that tumors were measured, the percent growth of each tumor was averaged with the other tumors in the same group to obtain a mean value of percent tumor growth. From days 5–14 post-treatment, One-way ANOVAs showed a significant difference between the groups (*p* = 0.001, days 7, 10, 12, and 14; *p* = 0.01 day 5; *n* = 10–13 mice). LSD post-hoc analyses showed significant differences (*p* < 0.05) between the FeSO_4_-PyED + Heat group and all other groups from days 12–14, and significant differences between the FeSO_4_-PyED + Heat and the Vehicle + Heat and Vehicle Only groups on days 5–10 (*p* < 0.05) (or for *p* < 0.1, between the FeSO_4_-PyED + Heat group and all other groups from days 7 to 14). Data from three experiments (2–5 mice per group per experiment).

**Figure 6.**
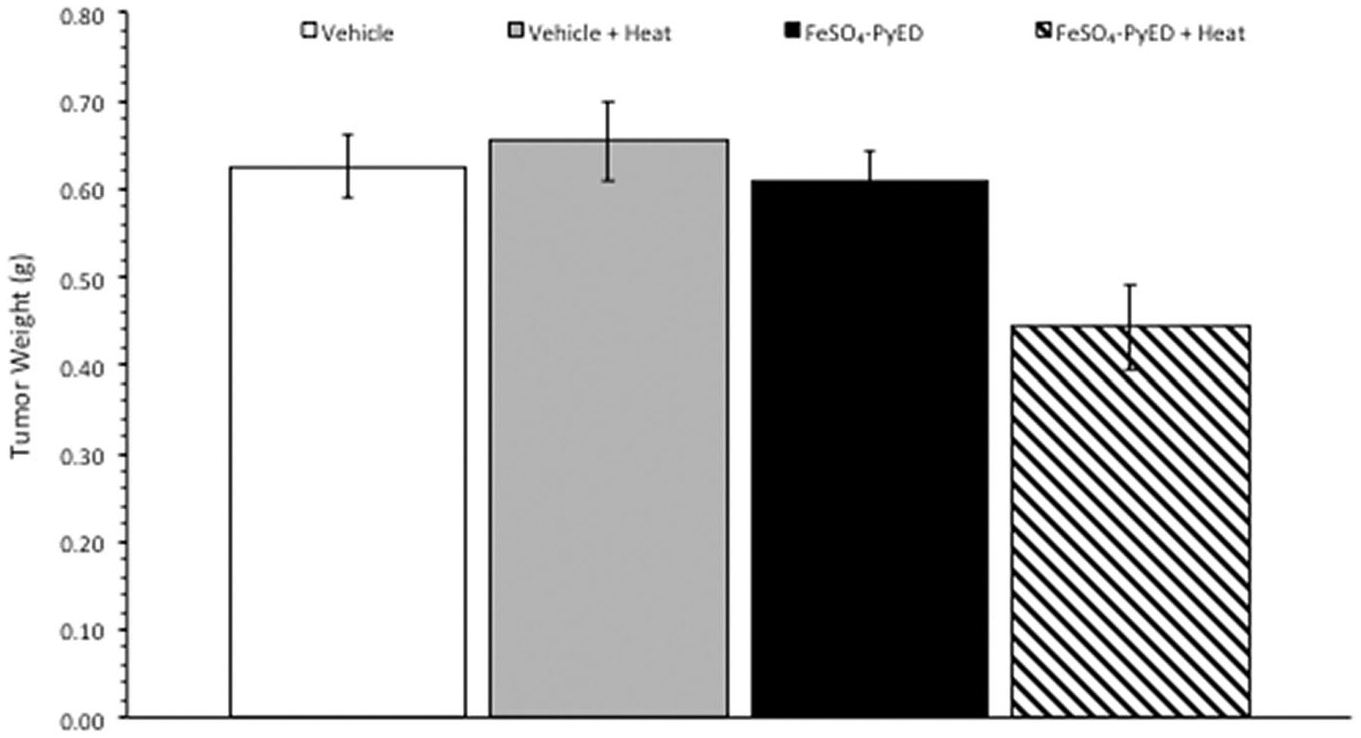
Treatment of C32 melanomas with FeSO_4_-PyED during heating inhibits tumor growth in a xenograft mouse model: Measurement of tumor weights. Tumor weights were determined on Day 14 after sacrificing mice that had been treated at either 33.5° or 42.5 °C with drug or vehicle as described in Figure 5. Tumors obtained from mice treated with FeSO_4_-PyED during hyperthermia treatment were significantly smaller than the tumors in all other groups. The *p* value from a One-way ANOVA was *p* = 0.002. LSD post-hoc tests showed that tumor weights in the FeSO_4_-PyED + heat group was significantly different from tumor weights in each of the other groups (vs. Vehicle, *p* = 0.003; vs. Vehicle + Heat, *p* = 0.001; vs. FeSO_4_-PyED, *p* = 0.004; *n* = 10–13). Data from three experiments as noted in Figure 5.

Since FeSO_4_-PyED induced significant apoptotic death in cultured human melanoma cells but not normal human melanocytes, we speculated that the compound might induce tumor cell killing or inhibition of tumor growth *in vivo*, with minimal or no significant effects on surrounding normal tissue. *In vivo* studies were performed using a C32 human melanoma tumor xenograft. This model was selected since melanomas have been treated previously with hyperthermia alone in the clinic, with curative intent, with well-defined heat doses having been successfully delivered to the tumor volume [37]. Tumors grown in hind limbs of 10–12 week old male NRG mice were treated with FeSO_4_-PyED or vehicle ~45 min prior to immersing the tumor-bearing hind limb for 1 h in 34° or 42.5 °C water baths (representing normothermic or hyperthermic conditions, respectively). When tumor volume was measured to determine the percent growth of each tumor for up to 14 days post-treatment, significant inhibition of tumor growth was observed in mice that received FeSO_4_-PyED treatment during 42.5 °C heating as determined by LSD post-hoc tests (see Figure 5). On Day 14 post-treatment, mice were euthanized, tumors were excised, and tumor weights determined for all treatment groups. Using a One-way ANOVA, we found that mice treated with FeSO_4_-PyED during heating had tumor weights that were significantly lower than tumors from the other treatment groups (*p* < 0.05) (Figure 6). No deleterious effects of hyperthermia or drug treatments, when administered individually or in combination, were observed with respect to normal tissue damage and ambulatory status; upon removal of tumors from the hind limb, no evidence of normal tissue necrosis was noted at the tumor periphery. On average, mice from all groups lost 6% of their body weight over the 14 days post-treatment, but there was not a significant difference between the groups in the percentage of weight lost (One-way ANOVA *p* = 0.646; data not shown).

The relative change in volume of individual tumors on day 14 post-treatment is plotted as a function of the initial tumor volume on day 0 of the experiment in Supplementary Figure 1. The overall average starting tumor volume for all animals in this study was 167 mm^3^. The average tumor volume for the control and each treatment group varied by less than 10% compared with the overall average. Mean tumor volumes for the Control (vehicle), Vehicle + Heat, FeSO_4_-PyED and FeSO_4_-PyED + Heat groups were 97%, 104%, 111%, and 99% of the overall mean tumor volume for all groups combined. Thus, there was a reasonably equal distribution of relatively small and large tumors within each group. While there was some variation in the sizes of individual tumors in the various groups at the beginning of the experiment, and smaller tumors generally grew faster than larger tumors in the Control (vehicle), Vehicle + Heat, and FeSO_4_-PyED groups, most of the tumors in the FeSO_4_+Heat group did not show large changes in tumor growth compared to the other groups, regardless of initial size.

## Discussion

In previous studies, we demonstrated that metallation can significantly alter the activation profiles of enediynes, and these observations led us to propose that metalloenediynes may prove to be more controllable, and therefore therapeutically more viable than their organic unmetallated counterparts [29]. Metalloenediynes can be synthesized at ambient temperature, but supraphysiological temperatures can be used to trigger cyclization on demand and initiate diradical H-atom abstraction from biological targets such as DNA [28,38,39]. Indeed, our recent initial characterization of a suite of novel metalloenediynes revealed that micromolar concentrations are generally nontoxic to tumor cells treated at 37 °C *in vitro*, but exposure to even slightly supra-physiological temperatures significantly enhances cytotoxicity [32]. In our current study, we further characterized the effects of one of these enediynes, FeSO_4_-PyED, *in vitro* and initiated experiments to assess the efficacy of the compound in inhibiting tumor growth *in vivo*.

Bergman cyclization from thermal activation of metalloenediynes results in diradical generation, and cyclization reactivity has been correlated with efficiency of degradation of supercoiled DNA *in vitro* [29]. In contrast, minimal degradation of DNA is observed in the presence of the cyclized analogs. Using the γ-H2AX assay, we previously have shown that hyperthermia enhances production of DSBs or results in the inhibition of DSB repair in cultured U-1 melanoma cells treated with FeSO_4_-PyED; thus, potentiation by hyperthermia of cell death induced by FeSO_4_-PyED is likely mediated via the creation of more initial DSBs or through alteration of the DNA damage repair response [32]. However, the association between Bergman cyclization and enhancement of cytotoxicity when cells are treated with metalloendiynes during hyperthermia treatment compared to treatment at 37 °C has been less clear. We hypothesized that enhancement of cytotoxicity after treatment at an elevated temperature is mediated through Bergman cyclization. Testing for a differential effect of the non-reactive cyclized analog, FeSO_4_-PyBD, on cell survival at physiological vs. supraphysiological temperatures could directly address this issue. A comparison of survival curves generated for U-1 cells with either FeSO_4_-PyED or FeSO_4_-PyBD, at 37 °C or 42.5 °C, revealed no difference in clonogenic survival for cells treated with either compound at 37 °C (Figure 2). However, heating at 42.5 °C during FeSO_4_-PyED treatment significantly enhanced cytotoxicity compared to treatment at 37 °C, whereas heating at 42.5 °C with FeSO_4_-PyBD did not. These data indicate that Bergman cyclization and formation of the diradical is wholly responsible for heat-induced enhancement of metalloenediyne cytotoxicity.

Enediynes have been referred to as radiomimetic; like IR, which may mediate its damage to DNA *via* free radical attack from the radiolysis of water molecules in the vicinity of DNA, enediyne-mediated damage to DNA occurs when the diradical abstracts hydrogen from deoxyribose [40]. Abstraction creates formation of a radical at the reacted carbon, which in turn may react with oxygen to form single or double strand breaks (DSBs), as well as abasic sites within short stretches of base pairs on opposite DNA strands which can also lead to breaks. IR can induce oxidative lesions in DNA that are closely-spaced and are referred to as locally multiply damaged sites [41]. Since FeSO_4_-PyED binds to DNA (Figure 3), it is attractive to speculate that metalloenediynes may induce clustered DNA damage species on opposing strands *via* metal-mediated radicals generated in the vicinity of the DNA to produce DSBs that result in lethality, similar to IR. Interestingly, hyperthermia potentiates the effects of IR, and this potentiating effect is due to inhibition of repair of IR-induced DSBs [42].

To date, all of our studies of the biological effects of metalloenediynes have utilized tumor cell lines [30–32]. Generally, no fundamental differences have been observed between the sensitivity of normal and tumor cells to hyperthermia [43], but sensitivity to chemotherapeutic agents can vary considerably. The effects of several chemotherapeutic agents are enhanced by hyperthermia, often due to increased drug influx or decreased efflux, which in turn may lead to greater intracellular drug accumulation and induction of more damage [3,4]. While hyperthermia appears to enhance metalloenediyne cytotoxicity *via* a different mechanism, a significant difference between the enhancement of killing of tumor cells compared to normal cells may be suggestive of intrinsic differences between tumor and normal tissue *in vivo*. If true, then the therapeutic gain observed after combination treatment with hyperthermia and metalloenediynes might be greater than treatment with either agent alone.

We chose to use PIG1 cells as a tool to study differences in cell killing between melanocytes and melanoma cells treated with FeSO_4_-PyED at 37° or 42.5 °C since PIG1 cells show extensive similarity to the original primary cells from which they were developed. They possess normal melanocytic properties and have been used to compare cell signaling between melanocytes and melanoma cells that may be related to metastatic potential [44], and to study the etiology of melanoma and pigmentary disorders.

Agents such as UV radiation and several chemotherapeutic agents that induce DNA strand breaks directly or indirectly are known to induce a moderate to strong apoptotic response in many melanoma lines and melanocytes [45]. Our previous studies suggested that apoptosis appears to be the primary mode of death induced by metalloenediynes once thermally activated in breast cancer and melanoma cells. Apoptosis was greatly enhanced when U-1 melanoma cells were treated with metalloenediynes under hyperthermic conditions. Treatment of U-1 cells with FeSO_4_-PyED during heating greatly increased the apoptotic fraction of cells 18–24 h later (32); also see Figure 4 for a representative data set]. However, in this study, apoptosis was not enhanced when PIG1 cells were treated with FeSO_4_-PyED at 42.5 °C. This led us to conclude that FeSO_4_-PyED has minimal effects on normal cells, even when administered during heating.

Using a melanoma xenograft model, we found that heating tumor-bearing hind limbs at 42.5 °C during FeSO_4_-PyED treatment resulted in an inhibition of tumor growth over a 14-day period post-treatment (Figure 5). The shape of a melanoma engrafted on the hind limb can vary between mice and even within the same mouse at different times post-treatment, and depth may not always be accurately estimated when calculating tumor volumes. Therefore, we also determined tumor weights of mice from each treatment group at the time of sacrifice on day 14 to confirm that tumor volume measurements enabled a valid comparison of tumor growth between treatment groups. Indeed, mice treated with FeSO_4_-PyED during heating yielded tumors that weighed significantly less than tumors from mice treated with vehicle, drug, or heat only (Figure 6). No normal tissue damage was observed.

In this study, the mean core temperature of mice with tumors that were not heated was 32.3 °C while the mean core temperature of mice with tumors that were heated was 37 °C. While there are likely to be physiological differences between a steady-state body temperature of 32.3° vs. 37 °C, it is important to note, however, that drug effects would be the result of the temperature of the tumor rather than the systemic temperature, since we have shown previously *in vitro* that there is no enhancement of FeSO_4_-PyED cytotoxicity noted at temperatures of 38.5 °C or lower [32]. It is possible that tumor perfusion could have increased in the mice with a systemic temperature of 37 °C; if this occurred, then some of the drug directly injected intratumorally may have been washed out, but that may have been offset by an increased delivery (to the tumor) of the drug that was systemically administered. Therefore, we do not believe that the systemic effects of, and differences in body temperature influenced the enhancement of drug effects on tumor growth.

Taken together, our studies suggest that there is a differential effect of combined drug + hyperthermia treatment between tumor and normal cells. Thus, enhancement of drug cytotoxicity in mice treated with FeSO_4_-PyED during heating using moderate hyperthermia or thermal ablation could potentially be confined to the heated tumor volume, sparing normal tissue from the common side effects observed from systemic administration of chemotherapy.

Moderate hyperthermia induces a myriad of cellular effects, and through vascular dilation and increases in perfusion, it can alter the tumor microenvironment. These changes can increase sensitivity of tumors when heat is administered with chemotherapy or radiation. Concurrent treatment with *moderate* hyperthermia and chemotherapeutic agents has shown promising results in phase III clinical trials [46–48]. However, clinical utilization of heat as an adjuvant to chemotherapy has not been widespread, because although drug cytotoxicity is often significantly potentiated *in vitro* by heating during drug treatment [37,49](often due to enhancement of drug delivery), most agents are very cytotoxic when administered systemically at normal body temperature. Heating at moderate temperatures is clinically achievable, and such treatments can increase response rates of locally advanced breast cancer when combined with Taxol and radiotherapy [37].

A drug, such as a metalloenediyne, which can be administered systemically or by direct delivery to a tumor and thermally activated in a *targeted manner* (e.g., using approaches like isolated limb infusion or perfusion) with limited or no toxicity to normal tissue would be clinically significant. Moderate hyperthermia, administered locally to a defined tumor volume (e.g., *via* radiofrequency current or ultrasound), could serve to target drug action by increasing tumor cell killing without causing systemic toxicity, thereby increasing the therapeutic gain *via localized drug activation*. Combining metalloenediyne chemotherapy with thermal ablation could also address an important problem associated with the latter modality: incomplete or heterogeneous destruction of the tumor. The success of thermal ablation (which can improve survival of patients with inoperable primary or metastatic disease and has become widely used in treating several tumors) is determined by the success in killing cells at the tumor margin [50–53]. During ablation, a significant temperature gradient may occur near the margin, such that the temperature at the tumor periphery may be similar to a moderate-temperature hyperthermia treatment. Treatment with metalloenediynes, which are activated by moderate heating, may prove to be an attractive strategy for enhancing the death of tumor cells at the periphery of the ablation zone that might normally survive sublethal temperatures and result in local recurrence [33]. Thus, we propose there is clinical potential for combining the use of metalloenediynes with thermal therapy in the treatment of some types of tumors.

## Supplementary Material

Supplementary material
