# Supplementary figures and images for: Characterization and initial demonstration of in vivo efficacy of a novel heat-activated metalloenediyne anti-cancer agent
Source: Int J Hyperthermia. Author manuscript; Available in PMC 2022 Oct 27. (PMC9612397; doi:10.1080/02656736.2021.2024280)

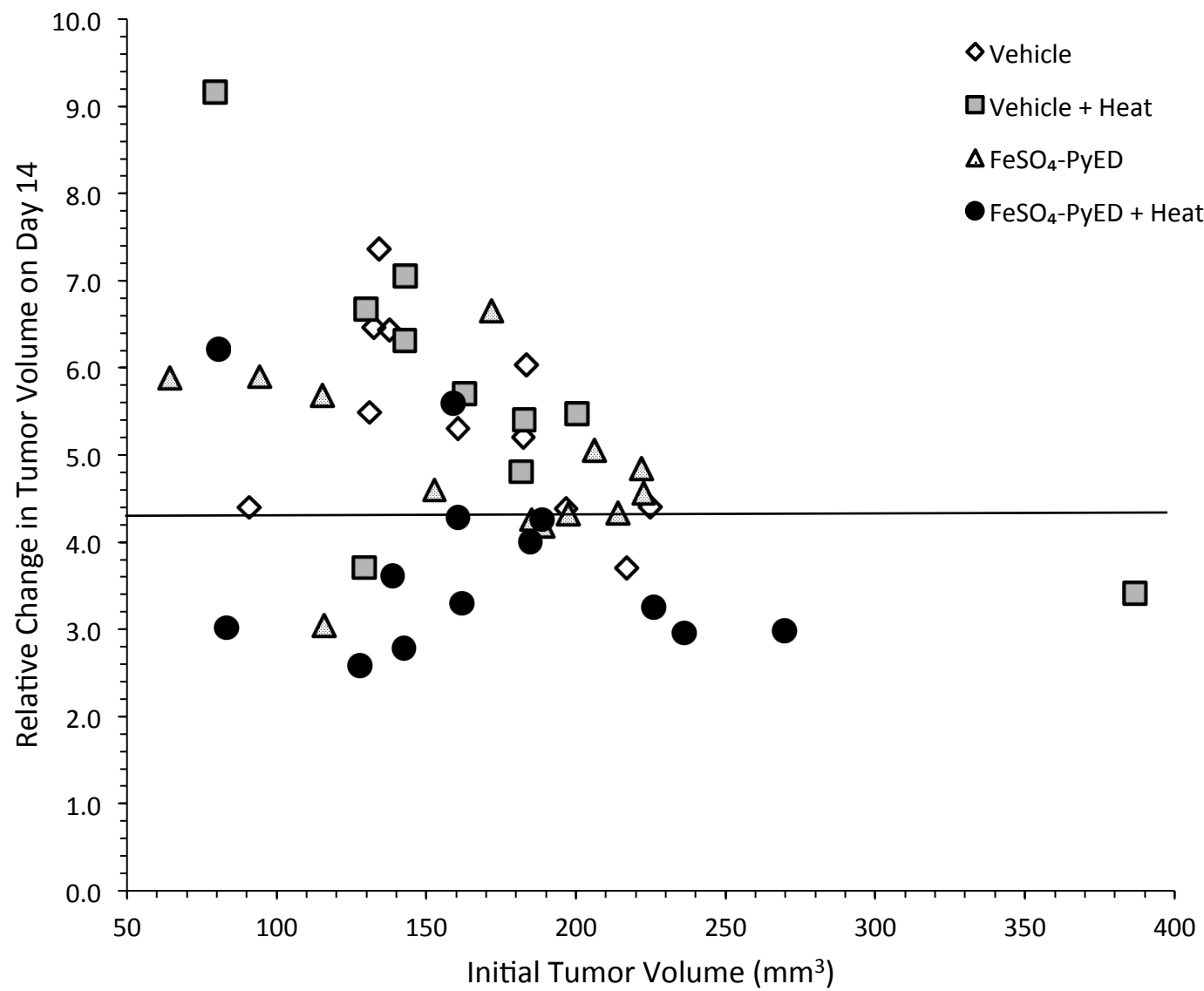

Supplement: Supplementary material [file NIHMS1839257-supplement-Supplementary_material.pdf]
